# Genomically matched therapy in refractory colorectal cancer according to ESMO Scale for Clinical Actionability of Molecular Targets: experience of a comprehensive cancer centre network

**DOI:** 10.1002/1878-0261.13444

**Published:** 2023-06-12

**Authors:** Núria Mulet Margalef, Carmen Castillo, Miguel Mosteiro, Xavier Pérez, Susana Aguilar, Fiorella Ruíz‐Pace, Marta Gil, Carmen Cuadra, José Carlos Ruffinelli, Mercedes Martínez, Ferran Losa, Gema Soler, Àlex Teulé, Roser Castany, Rosa Gallego, Andrea Ruíz, Elena Garralda, Elena Élez, Ana Vivancos, Josep Tabernero, Ramon Salazar, Rodrigo Dienstmann, Cristina Santos Vivas

**Affiliations:** ^1^ Medical Oncology Department Institut Català d'Oncologia L'Hospitalet de Llobregat Spain; ^2^ Badalona Applied Research Group in Oncology, B‐ARGO Spain; ^3^ Molecular Pre‐Screening Program Vall d'Hebron Institute of Oncology (VHIO) Barcelona Spain; ^4^ Oncology Data Science Group Vall d'Hebron Institute of Oncology (VHIO) Barcelona Spain; ^5^ Research Unit for Molecular Therapy of Cancer (UITM) Vall d'Hebron Institute of Oncology (VHIO) Barcelona Spain; ^6^ Department of Medical Oncology Vall d'Hebron Hospital Campus and Vall d'Hebron Institute of Oncology Barcelona Spain; ^7^ Cancer Genomics Group Vall d'Hebron Institute of Oncology (VHIO) Barcelona Spain; ^8^ Vall d'Hebron Hospital Campus and Institute of Oncology (VHIO) IOB‐Quiron, UVic‐UCC, CIBERONC Barcelona Spain; ^9^ Oncobell Program (IDIBELL), Facultat de Medicina i Ciències de la Salut Universitat de Barcelona CIBERONC Spain

**Keywords:** clinical trials, colorectal cancer, ESCAT, expanded genomic profiling, next‐generation sequencing

## Abstract

Efficiency of expanded genomic profiling (EGP) programmes in terms of final inclusion of patients in genomically matched therapies is still unknown. Fit patients with advanced and refractory colorectal cancer (CRC) were selected for an EGP programme. Next‐generation sequencing (NGS) analysis from formalin‐fixed paraffin‐embedded tumour samples was performed. The purpose was to describe the prevalence of genomic alterations defined by the ESMO Scale for Clinical Actionability of Molecular Targets (ESCAT), as well as the percentage of patients finally included in genomically guided clinical trials. In total, 187 patients were recruited. Mutational profile was obtained in 177 patients (10 patients were failure due to insufficient tumour sample), copy number alterations in 41 patients and fusions in 31 patients. ESCAT‐defined alterations were detected in 28.8% of the intention‐to‐analyse population. *BRAF* V600E was clustered in ESCAT I, with a prevalence of 3.7%, *KRAS* G12C and *ERBB2* amplification were clustered in ESCAT II, whose prevalence was 4.2% and 1.6%, respectively. Most alterations were classified in ESCAT III (mutations in *ERBB2*, *PIK3CA* or *FGFR* genes and *MET* amplification) and IV (mutations in *BRAF* non‐V600E, *ERBB3*, *FBXW7*, *NOTCH*, *RNF43*), with a single prevalence under 5%, except for *PIK3CA* mutation (9%). The final rate of inclusion into genomically guided clinical trials was 2.7%, including therapies targeting *BRAF* V600E or *RNF43* mutations in two patients each, and *ERBB2* mutation in one patient. In conclusion, EGP programmes in patients with advanced CRC are feasible and identify a subset of patients with potentially druggable genomic alterations. However, further efforts must be made to increase the rate of patients treated with genomically guided therapies.

## Graphical abstract

We have observed that an expanded genomic profiling programme in advanced colorectal cancer unveils druggable alterations in up to 30% of patients. However, the prevalence of these aberrations for single genes according to our programme is low, and the clinical benefit of their matched drugs is limited. Finally, our rate of inclusion in biomarker‐guided trials is under 3%.

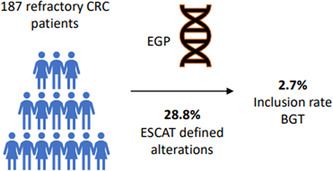

**Abbreviations**
AKT: AKT serine/threonine kinase
ALK: anaplastic lymphoma kinase
Anti‐EGFR: anti‐epidermal growth factor receptor
APC: adenomatous polyposis coli
ATM: ataxia telangiectasia mutated
BGT: biomarker‐guided trials
BRAF: v‐Raf murine sarcoma viral oncogene homologue B
CNA: copy number alterations
CRC: colorectal cancer
CTNNB1: catenin (cadherin‐associated protein), beta 1
ECOG: Eastern Cooperative Oncology Group
EGFR: epidermal growth factor receptor
EGP: expanded genomic profiling
ERBB: Erb‐b receptor tyrosin kinase
ESCAT: ESMO Scale for Clinical Actionability of Molecular Targets
ESMO: European Society for Medical Oncology
FBXW7: F‐box and WD repeat domain containing 7
FFPE: formalin fixed paraffin embedded
FGFR: fibroblast growth factor receptor
GNAS: guanine nucleotide binding protein (G protein) alpha stimulating
JAK: Janus kinase
KRAS: Kirsten rat Sarcoma virus
mCRC: metastatic colorectal cancer
MET: MET proto‐oncogene
MMR: mismatch repair system
MSI‐H/dMMR: microsatellite instability high, deficient mismatch repair
NGS: next‐generation sequencing
NOTCH: Notch (*Drosophila*) homologue
NRAS: neuroblastoma RAS viral oncogene homologue
NTRK: neurotrophic tyrosine kinase
PIK3CA: phosphatidylinositol 3‐kinase gene
POLE: polimerasa ε
PS: performance status
PTEN: phosphatase and tensin homologue
RET: Ret proto‐oncogene
RNF43: ring finger protein 43
SMAD4: SMAD family member 4
STK11: serine/threonine kinase 11
TP53: tumour protein P53

## 1 Introduction

Colorectal cancer (CRC) is one of worldwide leading causes of cancer death [1]. The prognosis of most patients with advanced disease has reached a plateau with the current standard of treatment based on chemotherapy plus biologic agents [2]. In the recent years, we have witnessed the irruption of the so‐called precision medicine with a continuous increase in genomic‐matched therapies that aim to improve patient's outcome [3]. In fact, metastatic CRC (mCRC) was one of the first tumours in which precision medicine was implemented through hotspot *KRAS‐NRAS* gene sequencing due to its negative predictive value of response to anti‐Epidermal Growth Factor (anti‐EGFR) antibodies [4, 5]. Beyond RAS status, other molecular subgroups have been more recently described in mCRC, like those harbouring *BRAF* V600E mutation, Microsatellite Instability High/Deficient Mismatch Repair (MSI‐H/dMMR), *POLE* mutation, *ERBB2* amplification, *MET* amplification or *NTRK* fusion, among others [6]. Since these aberrations are considered druggable and some of them even decisive in the continuum of care according to the latest ESMO Guidelines [2], it seems reasonable to determine them at some time point of advanced disease using Next‐Generation Sequencing (NGS) techniques. However, some issues must be considered. First, the incidence of these molecular alterations ranges from 1% to 8%, and the access to matched therapies (in the setting of clinical trials or not) is highly variable [6]. Second, the clinical value according to ESMO Scale for Clinical Actionability of molecular Targets (ESCAT) classification clearly differs between them (Table 1) [7, 8]. Determination of ESCAT I *RAS*/*BRAF* and Mismatch Repair System (MMR) status are already recommended per clinical guidelines in all patients with mCRC, and they do not require the upfront use of broad NGS panels [2, 9]. Third, expanded genomic profiling (EGP) must be executed in highly qualified centres, not only to ensure an accurate technique *per se*, but also for the proper interpretation of the results and accessibility to clinical trials with innovative targeted drugs [10]. Consequently, the cost‐efficiency of routine implementation of NGS in mCRC in terms of final access to drug‐matched therapies with direct impact on patient prognosis is still on debate. Indeed, ESMO does not support it, apart from molecular screening programmes for guiding clinical trials enrolment in reference centres [8].

**Table 1:** Definition of ESCAT levels and molecular alterations in CRC [7, 8].

ESCAT	Definition	Specific alterations in CRC
I	Validated in clinical trials	Mutations: *BRAF* V600E
		Fusions: *NTRK1*
		Others: MSI‐H/dMMR
		*RAS* WT
II	Responses in phase I/II/retrospective studies	Mutations: *KRAS* G12C Amplifications: *ERBB2*
III	Validated in malignancies different from the treated one	Mutations: *PIK3CA*, *ATM*, *AKT*, *FGFR*, *ERBB2*
		Amplifications: *MET*
		Fusions: *RET*, *ALK*
IV	Preclinical data	Mutations: *BRAF* Non – V600E, *ERBB3*, *FBXW7*, *NOTCH*, *RNF43*

In this work, a cohort of mCRC patients included in a EGP programme with therapeutic purposes has been analysed. The final aim was to describe the landscape of druggable alterations according to ESCAT, as well as the final inclusion in genomically guided clinical trials.

## 2 Materials and methods

Patients from Catalan Institute of Oncology who were included in an EGP programme between January 2015 and December 2020 and who met the following inclusion criteria were selected for the study cohort: (a) Refractory mCRC, defined by failure or intolerance to oxaliplatin, irinotecan, fluoropyrimidines, and antiangiogenics and anti‐EGFR, if both indicated; (b) Eastern Cooperative Oncology Group (ECOG) performance status (PS) 0 or 1; (c) No major comorbidities that could preclude participation in a clinical trial; (d) Availability of formalin fixed paraffin embedded (FFPE) sample, either from primary tumour or metastases. (e) Signed specific informed consent for tumour molecular profiling was approved by Local Ethics Committee (Bellvitge University Hospital. Reference PR046/15). The study methodologies conformed to the standards set by the Declaration of Helsinki.

Local molecular profile consisted of hotspot analysis of codons 12, 13, 59, 61, 117 and 146 of *KRAS* and *NRAS* genes, and codon 600 of *BRAF* gene by Real Time Polymerase Chain Reaction (RT‐PCR) if *RAS* wild‐type (WT). MSI‐H/dMMR phenotype was determined through PCR or by immunohistochemistry, following ESMO recommendations [11].

Next‐generation sequencing analyses were performed within the Vall d'Hebron Institute of Oncology (VHIO) Molecular Prescreening Program. Mutations were determined through a custom amplicon‐based NGS assay (MiSeq) covering genes relevant for cancer study (Table S1a,b). Copy number alterations (CNA) were assessed with a targeted copy number nCounter DNA panel of 44 genes (CNA nCounter). Finally, RNA sequencing was used to determine fusions through the nCounter RNA platform (NanoString). DNA mutations were aimed to be determined in the whole cohort, but the rest of molecular tests as per physician criteria after discussion in a molecular tumour board. The requirements of minimal tumour purity were of 20% for mutations assessment and fusion nCounter, and of 50% for CNA nCounter. The expected turnaround time for the entire analysis was between 2 and 3 weeks.

Summary tables of absolute and relative frequencies were used for descriptive analysis of categorical variables. Central value, average or median, and their values rank or 95% confidence intervals (CI) were applied for continuous variables.

Overall survival (OS) was defined as the time between the date of diagnosis of metastatic disease and the date in which cancer death was documented. Patients were censored at the date of the last contact in the absence of cancer death. OS was calculated using Kaplan–Meier curves. Statistical analyses were performed with ibm® spss® statistics V22 (Company IBM Corporation, Armonk, NY, USA).

## 3 Results

### 3.1 Cohort description

One hundred and eighty‐seven patients were included in the study cohort, whose baseline characteristics are described in Table 2. Median age at diagnosis was 61 years (range 27–79), 127 patients (68%) were male, left‐sided CRC prevailed in 144 patients (76.5%), as well as stage IV at diagnosis in 144 cases (66%). Local molecular tumour profile showed 91 (49%) *RAS* mutant tumours, 8 (4%) *BRAF* V600E tumours and 6 (3%) MSI‐H/dMMR tumours, although MMR status was not fully completed in the whole cohort. Median number of pre‐EGP treatments for advanced disease was 2. One, two and three or more lines of treatment had been previously administered to 12 (6%), 82 (44%) and 93 (50%) of patients, respectively. Regimens had included approved anti‐EGFR drugs and antiangiogenics at some point of advanced disease in 87 (46%) and 82 (44%) cases, respectively. Ten patients (5%) had been enrolled in clinical trials before NGS analysis, one of which was a biomarker‐guided trial (targeting *BRAF* V600E). The median OS of the cohort was 35.9 months (CI 95%, 32.3–39.6), after a median follow‐up of 39 months (3.13–138.8 months).

**Table 2:** Baseline characteristics of study cohort. UK, unknown.

Baseline characteristics		*N*: 187
		Total (%)
Age	61 years (27–79)	
Sex	Male	127 (68)
	Female	60 (32)
Sidedness	Left‐sided	144 (77)
	Right‐sided	43 (23)
Stage at diagnosis	I	4 (2)
	II	13 (7)
	III	46 (25)
	IV	124 (66)
*RAS*/*BRAF* status	*RAS* mutant	91 (49)^a^
	*BRAF* V600E	8 (4)^b^
	*RAS*/*BRAF* WT	76 (41)
	*RAS*/*BRAF* UK	12 (6)
MMR status	MSI‐H/dMMR	6 (3)^c^
	MSS /pMMR	166 (89)
	UK	15 (8)
Pre‐EGP lines of treatment	1	12 (6)
	2	82 (44)
	3 or more	93 (50)
Pre‐EGP regimens^d^	Based on anti‐EGFR	87 (46)
	Based on antiangiogenics	82 (44)
	Clinical trial	10 (5)

^a^ *NRAS* status was unknown in 1 patient.

^b^ *BRAF* status was unknown in 11 patients.

^c^ MMR status was unknown in 26 patients.

^d^ Therapeutic strategies administered at some point of the advanced disease.

### 3.2 NGS analysis procedures

**Fig. 1:**
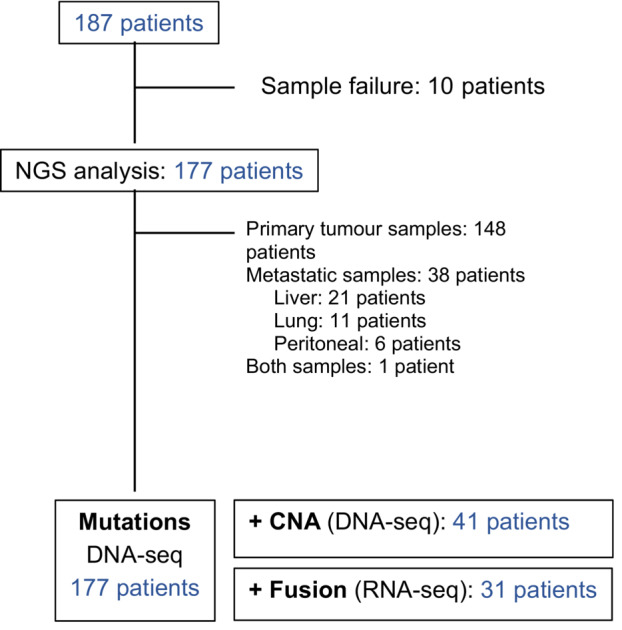
Flowchart of NGS analysis procedures. This figure illustrates the number of patients whose tumour samples could be analysed for each genomic aberration (mutation, CNA and fusion). The origin of tumour samples is also specified.

Procedures regarding NGS analysis are summarised in Fig. 1. Results were not available in 10 patients (5.3%) due to insufficient tumour representation in samples. No new biopsies for NGS purposes were performed. Primary tumour samples were used in 148 cases, metastasis sample in 38 cases, and both primary and metastases in one case. Mutations were finally assessed in 177 cases, CNA in 41 cases and fusions in 31 cases.

### 3.3 NGS analysis results

The NGS analysis results are summarised in Table 3, segregated by general cohort profile, sidedness (right‐sided *versus* left‐sided CRC) and local molecular profile according to *RAS* status (*RAS* wild‐type *versus RAS* mutant). Local *BRAF* and MMR status were not considered for the interpretation of the results because they were unknown in 11 and 15 patients, respectively (Table 2).

**Table 3:** Summary of NGS results (mutations and amplifications –*amp*‐) for the global cohort, according to sidedness and to local molecular profile. Each ESCAT molecular alteration is identified with the corresponding category superindex.

Gene	Global (*n* = 187, %)	Sidedness		RAS status	
		Right (*n* = 43, %)	Left (*n* = 144, %)	*RAS WT* (*n* = 95, %)	*RAS* mut (*n* = 91, %)
Mutations					
*APC*	112 (60)	23 (53.5)	89 (61.8)	52 (54.7)	56 (61.2)
*BRAF* V600E^I^	7 (3.7)	3 (7)	4 (2.8)	7	0
*BRAF* (other) ^IV^	4 (2.1)	2 (4.6)	2 (1.4)	4 (4.2)	0
*CTNNB1*	1 (0.05)	1 (2.3)	0	1 (1)	0
*ERBB2* ^III^	3 (1.6)	0	3 (2.1)	2 (2.1)	1 (1.1)
*ERBB3* ^IV^	4 (2.1)	1 (2.3)	3 (2.1)	1 (1)	3 (3.3)
*FBXW7* ^IV^	9 (4.8)	0	9 (6.2)	1 (1)	8 (8.8)
*FGFR1* ^III^	1 (0.05)	1 (2.3)	0	1 (1)	0
*FGFR2* ^III^	1 (0.05)	1 (2.3)	0	1 (1)	0
*FGFR3* ^III^	1 (0.05)	1 (2.3)	0	1 (1)	0
*GNAS*	4 (2.1)	0	4 (2.8)	1 (1)	3 (3.3)
*JAK*	1 (0.05)	1 (2.3)	0	0	1 (1.1)
*KRAS* G12C^II^	8 (4.2)	3 (7)	5 (3.5)	0	8 (8.8)
*KRAS* (other)	72 (38.5)	17 (39.5)	55 (38.2)	5 (5.3)	67 (73.6)
*MSH6*	2 (1)	1 (2.3)	1 (0.7)	0	1 (1.1)
*NOTCH1* ^IV^	1 (0.05)	0	1 (0.7)	1 (1)	0
*NRAS*	8 (4.2)	2 (4.6)	6 (4.2)	3 (3.2)	5 (5.5)
*PIK3CA* ^III^	29 (15.5)	10 (23.2)	19 (13.2)	9 (9.5)	19 (20.8)
*PTEN*	5 (2.7)	3 (7)	2 (1.4)	4 (4.2)	1 (1.1)
*RNF43* ^IV^	5 (2.7)	2 (4.6)	3 (2.1)	3 (3.2)	1 (1.1)
*SMAD4*	7 (3.7)	1 (2.3)	6 (4.2)	0	6 (6.6)
*STK11*	1 (0.05)	0	1 (0.7)	0	1 (1.1)
*TP53*	104 (55.6)	19 (44.2)	85 (59)	52 (54.7)	45 (49.4)
Other molecular alterations					
*ERBB2* amp^II^	3 (1.6)	1 (2.3)	2 (1.4)	3 (3.2)	0
*MET* amp^III^	2 (1)	0	2 (1.4)	2 (2.1)	0

Considering the intention‐to‐analyse population of 187 cases, DNA‐seq did not show any alteration in 10 cases and no gene fusions were identified. The most frequent mutated genes were *APC* (112, 60%), *TP53* (104, 55.6%), *RAS* (88, 46.9%) and *PIK3CA* (29, 15.5%). Intriguingly, some discrepancies were observed between local determination and NGS analysis regarding *RAS*. In previously reported as *RAS* wild‐type per local assessment, NGS analysis revealed *KRAS* and *NRAS* mutations in five and three patients, respectively. Tumour sample origin in which local PCR and NGS were performed was different in only one of them. Influence of anti‐EGFR treatment did not justify these findings in any case, because it was administered to patients after tumour sample collection. Conversely, *RAS* was mutated per local PCR in three cases, unlike NGS, even though tumour sample origin was the same for both analyses in all cases. Regarding *BRAF* gene, one tumour harboured V600E via local assessment, in contrast to NGS result; samples used for both procedures were from different origin.

The prevalence of the remaining molecular alterations including mutations and amplifications was under 5% (Table 3). However, some exploratory trends in the rate of ESCAT defined molecular alterations could be observed when sidedness and local molecular profile were considered. ESCAT I: *BRAF* V600E prevailed in right‐sided CRC (7% *versus* 2.8% in left‐sided CRC). ESCAT II: *ERBB2* amplification was identified only in *RAS* wild‐type patients (3.2%) and *KRAS* G12C prevailed in right‐sided CRC (7% *versus* 3.5% in left‐sided CRC). ESCAT III: *ERBB2* mutation was only found in left‐sided CRC (2.1%). *PIK3CA* mutation, although much more frequent in *RAS* mutant population (19 out of 91, 20.8%), could only be considered as druggable in *RAS* wild‐type population (9 out of 95, 9.5%). *FGFR*1‐3 mutations were only found in right‐sided CRC (2.3% each gene), contrary to *MET* amplification (1.4%), that additionally was only observed in *RAS* wild‐type population. ESCAT IV: non‐V600E *BRAF* mutations prevailed in right‐sided CRC (4.6% *versus* 1.4% in left‐sided CRC), like *RNF43* (4.6% *versus* 2.1%), and contrary to *FBXW7* mutations (9 out of 187, only observed in left‐sided CRC). Regarding local *RAS* status, *FBXW7* mutations prevailed in *RAS* mutant population (8.8% *versus* 1% in *RAS* wild‐type population). Genomic alterations in MSI‐H/dMMR patients were classified in ESCAT III and IV (Table S2).

### 3.4 Inclusion in biomarker‐guided clinical trials

A total of 58 ESCAT defined druggable alterations (Table 3) in 54 patients were observed across the cohort, since two druggable alterations coexisted in four patients: *BRAF* V600E—*FGFR2* mutation, *MET* amplification—*RNF43* mutation, *FGFR1* mutation—*RNF43* mutation and *BRAF* V600E—*RNF43* mutation.

**Fig. 2:**
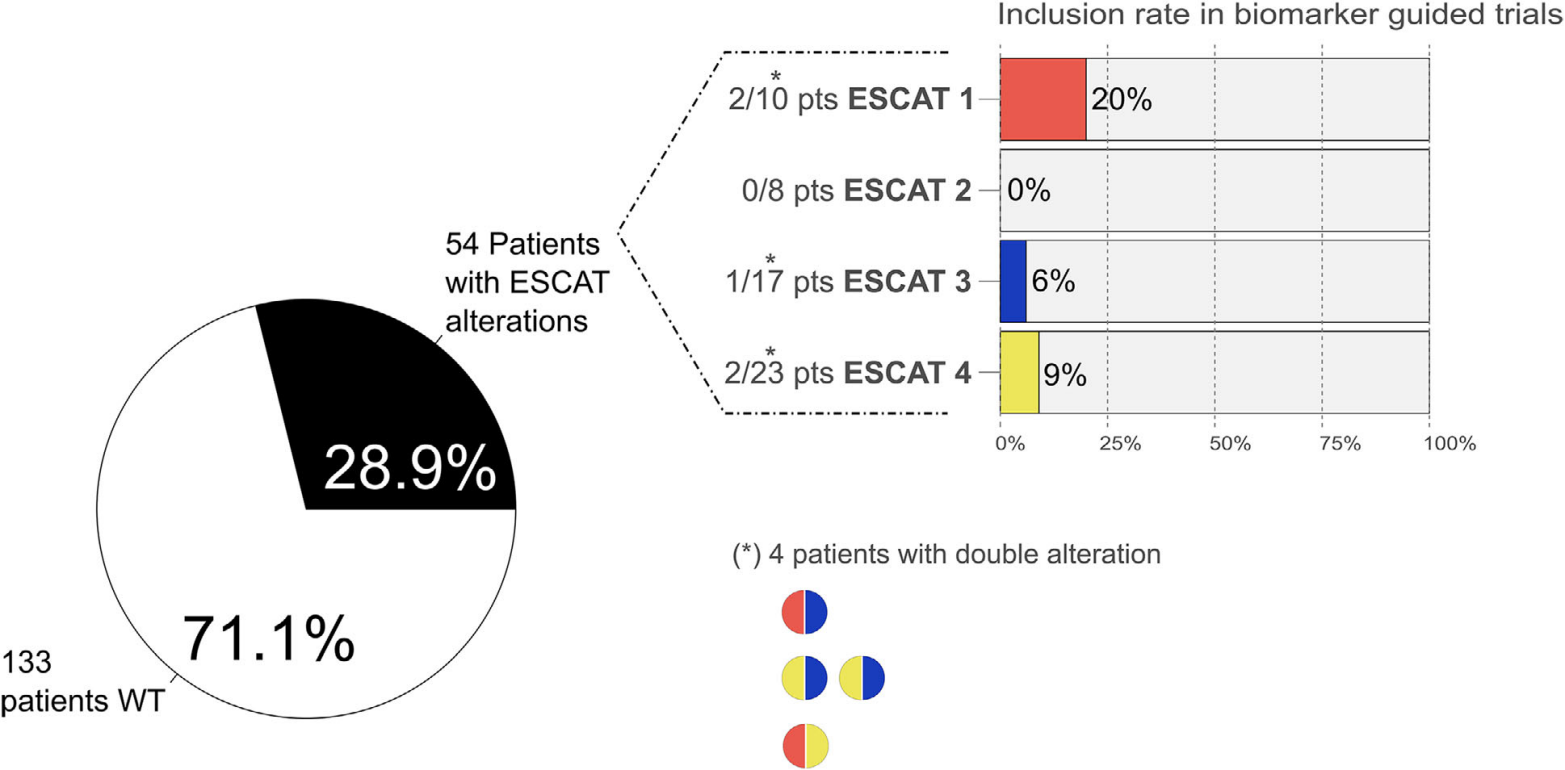
Prevalence of patients with ESCAT alterations and inclusion rate in BGT per ESCAT level. Patients WT in the figure refer to those without ESCAT alterations detected in tumour tissue.

Five patients out of 54 with druggable alterations were included in biomarker guided trials (trials targeting *BRAF* V600E in two patients, trials targeting *RNF43* mutation in two patients and *ERBB2* mutation in one patient), and one of them was included in two trials consecutively (patient whose tumour harboured *BRAF* V600E and *RNF43* mutation) (Fig. 2). Thirteen patients received best supportive care because of clinical deterioration driven by hepatic failure due to liver metastases, sepsis, bowel obstruction, pulmonary embolism, acute gastrointestinal bleeding and diagnosis of dementia, and 36 were treated with standard of care or with non‐biomarker‐guided trials (BGT) because of the lack of slots in biomarker‐guided ones. All but one patient with MSI‐H/dMMR tumours were treated with immune checkpoint inhibitors in the setting of non‐BGT.

As a result, and considering the intention‐to‐analyse population of 187 patients, ESCAT druggable alterations were observed in 28.9% of patients, but the final inclusion rate in BGT was 2.7% (5 out of 187 patients) (Fig. 2). However, after ruling out *BRAF* V600E and *KRAS* G12C since these alterations can be identified per local PCR test, ESCAT druggable alterations were observed in 20.1% of patients and 2.1% could be finally included in BGT.

## 4 Discussion

We report a cohort of 187 heavily pretreated mCRC patients (50% had previously received three or more lines of treatment) included in an NGS programme for therapeutic purposes. As expected, left‐sided CRC prevailed in more than 2/3 of cases, and about 50% of tumours were *RAS* mutant. The prevalence of *BRAF* V600E locally determined was 4%, slightly lower than predicted [2].

Considering NGS analysis, the results were not available in 10 out of 187 patients due to sample failure. This rate is lower than the 30% reported in other pan‐cancer studies [12, 13], partly justified by the relatively large amount of tumour content in CRC biopsies and surgical specimens, compared with other malignancies. In this sense, NGS analysis allows the evaluation of multiple biomarkers with the time frame and amount of available biological material.

Next‐generation sequencing results showed mutations in *APC*, *TP53*, *RAS* and *PIK3CA* as the most prevalent events, as described in other series [14, 15], all of them related with CRC carcinogenesis [16]. Of note, discordance in *RAS*/*BRAF* status between NGS and local PCR occurred in 12 out of 187 patients, probably explained by molecular heterogeneity in two cases (samples assessed were different in each technique), and by technical assay sensitivity issues in the rest [17]. Regarding ESCAT druggable alterations, they were identified in 28.9% of patients. Among them, ESCAT II – *ERBB2* amplification was observed in 1.6% of patients, and the remaining aberrations belong to ESCAT III and ESCAT IV categories with an individual prevalence below 5%, aligned with previous reports [10, 16]. The final inclusion rate in BGT was below 3%. Despite this low rate of inclusion, this is in line with previous data [18], highlighting that the possibility of targeted drug treatment, either under clinical trial or in routine practice, is clearly associated with NGS study's timeframe.

This work has some limitations. There was a bias in the selection of patients because only those with ECOG PS suitable for clinical trial inclusion were included. Therefore, the representation of less prevalent molecular profiles associating druggable aberrations in CRC, like right‐sided and *RAS*/*BRAF* wild‐type [19], is limited. Regarding *RAS* mutant population, that accounted for almost 50% of the cohort, descriptive data in Table 3 show lesser prevalence of ESCAT alterations (statistic comparisons have not been performed because of the absolute low number of cases with molecular aberrations). Thus, the cost‐effective balance of including *RAS* mutant tumours in EGP could be argued, although updated research could change this assumption, like the preliminary data of trastuzumab deruxtecan activity in this subset of patients [20]. Second, the analyses were performed mainly on primary tumours (Fig. 1), and no new metastatic biopsies were obtained for NGS testing. Thus, the molecular heterogeneity of mCRC [21] could have not been fully captured. However, the rate of concordance between primary samples and metastases in terms of NGS results is about 70–80% according to other series [22], and a recent pan‐tumour work concluded that prospective genomic analysis using Whole Exome Sequencing do not reveal new biomarkers in more than 90% of cases compared with baseline ones [23]. In *RAS* WT population treated with anti‐EGFR, molecular aberrations resulting from therapeutic pressure were not analysed [24, 25], since rebiopsy was not mandatory for the inclusion in the EGP, and it did not include liquid biopsy. Nonetheless, it should be considered that the EGP started in 2015, before the publication of relevant data in the field. Additionally, only 30% of the patients received anti‐EGFR‐based therapy as last line before being included in the EGP. Considering the decay of anti‐EGFR resistant clones after a 4–6‐month anti‐EGFR washout period [26], we could speculate the low efficiency of molecular retesting for this purpose. Third, NGS analyses were performed using a small panel, and fusions and CNA were not determined in the whole cohort, precluding this the identification of some drug biomarkers, like tumour mutational burden [27]. In addition, due to unknown MMR status 15 out of 187 patients, the information about the presence of MSI‐H/dMMR phenotype was not complete. These patients were included at the beginning of recruitment period when the aforementioned phenotype was not yet considered as ESCAT I alteration.

In conclusion, EGP programmes in mCRC are feasible in a reference centre and useful for identifying druggable alterations, although the inclusion rate in BGT is still very low. With the current implementation of EGP programmes in the daily practice, near‐future efforts must be focussed on: (a) reducing tissue and economical costs to guarantee equity; (b) improving the BGT portfolio to increase the therapeutic efficiency of NGS tests; (c) reshaping NGS panels periodically to cover emerging biomarkers according to basal CRC molecular profile; (d) redefining the decisive instant to perform NGS to capture the biological evolution of mCRC under therapeutic pressure; and (e) implementing liquid biopsy as an alternative to tissue when indicated.

## 5 Conclusions

Expanded genomic profiling programmes are feasible and unveil druggable alterations ESCAT I to IV in up to 30% of advanced CRC. However, the prevalence of druggable alterations for a given gene in mCRC is less than 5% (apart from *PIK3CA* mutations) and they mostly belong to ESCAT III and IV, although this classification could be variable over time. The rate of inclusion in BGT related with NGS programmes is under 3%, but it is highly dependent on clinical trials portfolio.

## Conflict of interest

Ana Vivancos has served in a consultant or advisory role for Merck, S.L., Madrid, Merck Serono and Sysmex. Cristina Santos Vivas receives travel and academic grants from Amgen and Merck, and advisory board fees from Amgen and Sanofi‐Aventis. Elena Élez declares personal financial interest for consulting or advisory roles or honoraria, travel grants, and research grants from Amgen, Bayer, Hoffman‐La Roche, Merck Serono, Sanofi, Pierre Fabre, MSD, Organon, Novartis, and Servier; institutional financial interest in the form of financial support for clinical trials or contracted research for Amgen, Array Biopharma, AstraZeneca Pharmaceuticals, BeiGene, Boehringer Ingelheim, Bristol Myers Squibb, Celgene, Debiopharm International, F Hoffmann‐La Roche, Genentech, HalioDX SAS, Hutchison MediPharma International, Janssen‐Cilag, MedImmune, Menarini, Merck Health KgAA, Merck Sharp & Dohme, Merus NV, Mirati, Novartis Farmacéutica, Pfizer, Pharma Mar, Sanofi Aventis Recherche & Développement, Servier, and Taiho Pharma USA. Elena Garralda: Research: Novartis/Roche/Thermo Fisher/AstraZeneca/Taiho/BeiGene Consultant‐Advisor: Roche/Genentech – F. Hoffmann/La Roche – Ellipses Pharma – Neomed Therapeutics1 Inc – Boehringer Ingelheim – Janssen Global Services – SeaGen – TFS – Alkermes – Thermo Fisher – Bristol‐Mayers Squibb – MabDiscovery – Anaveon – F‐Star Therapeutics – Hengrui. Speakers Bureau: Merck Sharp & Dohme/Roche/Thermo Fisher/Lilly/Novartis. Clinical Trials PI or Co‐PI (Institution): Agios Pharmaceuticals – Amgen – Bayer – Beigene USA – Blueprint Medicines – BMS – Cellestia Biotech – Debiopharm – F. Hoffmann La Roche Ltd – Forma Therapeutics – Genentech Inc – Genmab B.V. – GSK – Glycotope Gmbh – Incyte Biosciences – Incyte Corporation – ICO – Kura Oncology Inc – Lilly, S.A – Loxo Oncology Inc – Macrogenics Inc – Menarini Ricerche Spa – Merck, Sharp & Dohme de España, S.A – Nanobiotix, S.A – Novartis Farmacéutica, S.A – Pfizer, SLU – Pharma Mar, S.A.U – Pierre Fabre Medicament – Principia Biopharma Inc. – Psioxus Therapeutics Ltd – Sanofi – Sierra Oncology, Inc – Sotio A.S – Symphogen A/S. Josep Tabernero reports personal financial interest in form of scientific consultancy role for Array Biopharma, AstraZeneca, Bayer, Boehringer Ingelheim, Chugai, Daiichi Sankyo, F. Hoffmann‐La Roche Ltd, Genentech Inc, HalioDX SAS, Hutchison MediPharma International, Ikena Oncology, Inspirna Inc, IQVIA, Lilly, Menarini, Merck Serono, Merus, MSD, Mirati, Neophore, Novartis, Ona Therapeutics, Orion Biotechnology, Peptomyc, Pfizer, Pierre Fabre, Samsung Bioepis, Sanofi, Scandion Oncology, Scorpion Therapeutics, Seattle Genetics, Servier, Sotio Biotech, Taiho, Tessa Therapeutics and TheraMyc. Stocks: Oniria Therapeutics and also educational collaboration with Imedex/HMP, Medscape Education, MJH Life Sciences, PeerView Institute for Medical Education and Physicians Education Resource (PER). Núria Mulet Margalef receives travel and academic grants from Amgen, Merck and Roche. Ramon Salazar has served in a consultant or advisory role for Amgen, Merck, S.L., Madrid, Roche Dx and research funding for Roche Dx. Rodrigo Dienstmann has an advisory role at Roche and Boehringer‐ Ingelheim, has received a speaker’ s fee from Roche, Ipsen, Amgen, Sanofi, Servier, Libbs, Merck Sharp & Dohme and further received direct research funding from Merck and Pierre Fabre. José Carlos Ruffinelli has received travel grants from Merck and MSD and a speaker’ s fee from Amgen. The other authors have declared no conflicts of interest.

## Author contributions

CS, RS, RD and NMM have designed the work. NMM, CCa, MM, AR and CS have collected clinical data. XP, FR‐P and RD have performed statistical analysis and tables. MMV, CCu, JCR, FL, GS, AT, RC and RG have selected the patients for NGS programme. EG and EE have treated the patients in clinical trials. SA, AV, JT and RD have developed and supervised the NGS programme. All the authors have contributed to the manuscript design and they have approved the final version.

### Peer review

The peer review history for this article is available at https://www.webofscience.com/api/gateway/wos/peer‐review/10.1002/1878‐0261.13444.

## Supporting information


**Table S1.** Amplicon‐based NGS assays.
**Table S2.** Mutational genomic profile of MSI‐H/dMMR tumours.

## Data Availability

All the data supporting the work are available in the manuscript.
