# Supplementary material for: Genomically matched therapy in refractory colorectal cancer according to ESMO Scale for Clinical Actionability of Molecular Targets: experience of a comprehensive cancer centre network
Source: Mol Oncol. 2023 Jun 12;17(9):1908–16. doi: 10.1002/1878-0261.13444 (PMC10483603; doi:10.1002/1878-0261.13444)
Supplement: Supplementary file 1 — Table S1. Amplicon‐based NGS assays. Table S2. Mutational genomic profile of MSI‐H/dMMR tumours. [file MOL2-17-1908-s001.docx]

SUPPLEMENTARY DATA

**Supplementary Table 1. Amplicon-based NGS assays.**

a) Panel used from January 2015 until May 2018

| ABL1 | ERBB3 | IDH1 | NF2 | SMAD4 |
| --- | --- | --- | --- | --- |
| AKT1 | ESR1 | IDH2 | NOTCH1 | SMARCB1 |
| AKT2 | FBXW7 | JAK1 | NOTCH4 | SRC |
| AKT3 | FGFR1 | JAK3 | NRAS | STK11 |
| ALK | FGFR2 | KIT | PDGFRA | TP53 |
| APC | FGFR3 | KRAS | PIK3CA | VHL |
| BRAF | FGFR4 | MAG | PIK3R1 | ZNRF3 |
| CDH1 | FLT3 | MAP2K1 | PIK3R5 |  |
| CDKN2A | GATA1 | MET | PTEN |  |
| CSF1R | GNA11 | MLH1 | RB1 |  |
| CTNNB1 | GNAQ | MPL | RET |  |
| EGFR | GNAS | MSH6 | RNF43 |  |
| ERBB2 | HRAS | MYC | RUNX1 |  |

b)Panel used from May 2018 onwards (NOTCH 2 and NOTCH 3 were added, and FGFR4 was removed, in order to increase cancer origin specificity)

| ABL1 | EGFR | GNAQ | MET | PDGFRA | STK11 |
| --- | --- | --- | --- | --- | --- |
| AKT1 | ERBB2 | GNAS | MLH1 | PIK3CA | TP53 |
| AKT2 | ERBB3 | HRAS | MPL | PIK3R1 | VHL |
| AKT3 | ESR1 | IDH1 | MSH6 | PIK3R5 | RNF43 |
| ALK | FBXW7 | IDH2 | MYC | PTEN | ZNRF3 |
| APC | FGFR1 | JAK1 | NF2 | RB1 |  |
| BRAF | FGFR2 | JAK3 | NOTCH1 | RET |  |
| CDH1 | FGFR3 | KIT | NOTCH2 | RUNX1 |  |
| CDKN2A | FLT3 | KRAS | NOTCH3 | SMAD4 |  |
| CSF1R | GATA1 | MAG | NOTCH4 | SMARCB1 |  |
| CTNNB1 | GNA11 | MAP2K1 | NRAS | SRC |  |

| Patient | ESCAT I | ESCAT 2 | ESCAT III | ESCAT IV | Non-ESCAT alterations | Analysis |
| --- | --- | --- | --- | --- | --- | --- |
| 1 | RAS WT |  | FGFR3 |  | CTNNB1 |  |
| 2 |  |  | PIK3CA | FBXW7 | APC |  |
| 3 |  |  |  |  | APC, MSH6 |  |
| 4 | RAS WT |  |  |  | TP53, MSH6 | FUSION |
| 5 | RAS WT |  | FGFR1 | RNF43 |  | FUSION |
| 6 | RAS WT |  | PIK3CA |  |  | CNA FUSION |

Supplementary Table 2. Mutational genomic profile of MSI-H/dMMR tumours, according to ESCAT classification. The column “Analysis” describes if fusions and copy number alterations were also assessed.
